# Capturing Neuroplastic Changes after iTBS in Patients with Post-Stroke Aphasia: A Pilot fMRI Study

**DOI:** 10.3390/brainsci11111451

**Published:** 2021-10-31

**Authors:** Shuo Xu, Qing Yang, Mengye Chen, Panmo Deng, Ren Zhuang, Zengchun Sun, Chong Li, Zhijie Yan, Yongli Zhang, Jie Jia

**Affiliations:** 1 Department of Rehabilitation Medicine, Huashan Hospital, Fudan University, Shanghai 200040, China; xus20@fudan.edu.cn (S.X.); 07301010208@fudan.edu.cn (Q.Y.); babybreathks@126.com (M.C.); 2 Department of Rehabilitation Medicine, Jingan District Central Hospital Affiliated to Fudan University, Shanghai 200040, China; xyz3325127@163.com; 3 Department of Rehabilitation Medicine, Changzhou Dean Hospital, Changzhou 213000, China; zr2003@163.com; 4 Sichuan Bayi Rehabilitation Center, Affiliated Sichuan Provincial Rehabilitation Hospital of Chengdu University of TCM, Chengdu 610075, China; mikezsun@outlook.com; 5 Faculty of Sport and Science, Shanghai University of Sport, Shanghai 200040, China; lichongsus@163.com; 6 The Third Affiliated Hospital, Xinxiang Medical University, Xinxiang 453003, China; yzj2020mail@126.com; 7 Institute of Rehabilitation, Fujian University of Traditional Chinese Medicine, Fuzhou 350122, China; islilyong@163.com

**Keywords:** stroke, aphasia, iTBS, fMRI, neuroplasticity, rehabilitation

## Abstract

Intermittent theta-burst stimulation (iTBS) is a high-efficiency transcranial magnetic stimulation (TMS) paradigm that has been applied to post-stroke aphasia (PSA). However, its efficacy mechanisms have not been clarified. This study aimed to explore the immediate effects of iTBS of the primary motor cortex (M1) of the affected hemisphere, on the functional activities and connectivity of the brains of PSA patients. A total of 16 patients with aphasia after stroke received iTBS with 800 pulses for 300 s. All patients underwent motor, language, and cognitive assessments and resting-state functional MRI scans immediately before and after the iTBS intervention. Regional, seed-based connectivity, and graph-based measures were used to test the immediate functional effects of the iTBS intervention, including the fractional amplitude of low-frequency fluctuation (fALFF), degree centrality (DC), and functional connectivity (FC) of the left M1 area throughout the whole brain. The results showed that after one session of iTBS intervention, the fALFF, DC, and FC values changed significantly in the patients’ brains. Specifically, the DC values were significantly higher in the right middle frontal gyrus and parts of the left parietal lobe (*p* < 0.05), while fALFF values were significantly lower in the right medial frontal lobe and parts of the left intracalcarine cortex (*p* < 0.05), and the strength of the functional connectivity between the left M1 area and the left superior frontal gyrus was reduced (*p* < 0.05). Our findings provided preliminary evidences that the iTBS on the ipsilesional M1 could induce neural activity and functional connectivity changes in the motor, language, and other brain regions in patients with PSA, which may promote neuroplasticity and functional recovery.

## 1. Introduction

Patients with post-stroke aphasia (PSA) often have impaired upper extremity (UE) motor and cognitive function [1,2,3]. Non-invasive brain stimulation (NIBS), specifically transcranial magnetic stimulation (TMS) and transcranial direct current stimulation (tDCS), are emerging as promising NIBS modalities in treating the language and motor dysfunction of stroke patients [4,5,6,7,8]. Intriguingly, not only have the NIBS targeting the “motor” or “language” areas been proven beneficial for improving the patients’ motor or language performances, respectively [2], but recent studies have also reported that NIBS targeting the motor area (i.e., M1) could also improve the language function of the PSA patients [9]. As the M1 area is far easier to be located than classical language areas such as Broca’s area, this finding highlighted a potentially practical way to “kill two birds with one stone”, in treating the language and motor impairments of patients with stroke. However, although the structural and functional connectivity have been identified between the brain’s language and motor areas in healthy volunteers and the patients with various neurological disorders [10,11,12,13], it is still not clear if and how the NIBS targeting the M1 would affect the language function via these connections. Therefore, we conducted this preliminary functional MRI study to investigate the immediate brain functional effects of the left M1 stimulation with intermittent theta-burst stimulation (iTBS), which is a popular TMS method, in treating PSA using resting-state functional magnetic resonance (fMRI) images.

Intermittent theta-burst stimulation (iTBS) is a popular new TMS intervention paradigm, with a high within burst frequency usually at 50 Hz and a between burst frequency usually at 5 Hz. With seconds of intervals between trains of intervention, it has been proposed to be able to induce excitatory effects in the area of stimulation [14,15,16,17]. A standard iTBS session usually takes about three minutes to apply, and this time duration is easier for the patients to cooperate. This quick application is a major advantage of iTBS that makes it increasingly applied and reported in treating patients with various neurological disorders including stroke [17,18,19]. iTBS have been reported to be able to alter cortical excitability at the site of stimulation, as well as in its surrounding and even connected remote regions, [20]. Such effects have been also reported in other TMS paradigms. For example, Wang et al. [21] revealed the enhanced cortico-hippocampal functional connectivity (FC) by multi-session excitatory TMS over the lateral parietal cortex. Hawco et al. [22] reported spread TMS-induced cortical changes that were related to the FC between the stimulated site and salience network.

Interestingly, evidence from behavioral brain imaging and brain stimulation studies suggests that primary motor cortex (M1) stimulation by tDCS may represent a promising and clinically feasible approach to enhance language therapy outcomes in post-stroke aphasia [9]. A few studies have also found that repetitive TMS stimulating area M1 can improve speech functions such as verbal fluency and naming to varying degrees, as well as enhance cognitive function to some extent [23,24,25]. However, there is little research on whether iTBS stimulation of M1 in the affected cortex induces changes in the brains after stroke. Therefore, whether and how the iTBS targeting M1 would induce brain functional changes in PSA patients remains to be investigated.

Brain functional changes following iTBS can be detected using a series of non-invasive imaging and electrophysiological techniques [22,24,26]. In this study, we used resting-state fMRI, which is one of the most popular brain functional imaging methods, to measure the spontaneous neural activities of the brain, with a coverage of the whole cerebral cortex and subcortical structures at a millimeter-level resolution. Previous studies have proved that resting-state fMRI could reveal the spontaneous neural activities and functional connectivity patterns of the brain, which partially reflected the structural connectivity, and correlated with the brain activation pattern and behavioral performances under various task conditions [27,28]. In addition, the “resting-state” does not require the subject to perform specific tasks, which could be easier for patients to cooperate and facilitate comparisons across studies [29,30]. As iTBS and other TMS paradigms have been reported to be able to induce both regional neural activity and functional connectivity changes [31], we included both regional- and connectivity-based metrics to measure the functional changes induced by the M1 iTBS. Specifically, we used the fractional amplitude of low-frequency fluctuation (fALFF) [32] to reflect the regional spontaneous neural activity, while seed-based functional connectivity analyses were used to measure the functional dependence between the M1 and other regions in the brain; and we also adopted the degree centrality (DC), a graph theory-based metrics [33], to measure the relative importance of each region in the brain functional network [34].

Therefore, in our study, we used iTBS to stimulate the left M1 hand area, which is in the affected brain hemisphere of our patients with PSA, and observed its immediate effects on brain activity, using the resting-state fMRI data. This preliminary study aimed to explore the potential therapeutic mechanisms of motor area iTBS for treating patients with PSA.

## 2. Materials and Methods

### 2.1. Participants

Patients with post-stroke aphasia were recruited from Huashan Hospital, Fudan University. The inclusion criteria were as follows: (i) aged 18 or older; (ii) native Chinese speakers; (iii) clinical diagnosis as ischemic or hemorrhagic stroke at hospital discharge from the neurological department, confirmed with computed tomography (CT) or magnetic resonance images (MRI); (iv) able to complete the study; (v) aphasia quotient below 93.8 assessed by the Chinese version of western aphasia battery (WAB). The exclusion criteria were as follows: (i) previous stroke; (ii) severe psychiatric condition; (iii) epilepsy, (iv) other neurological disorders, such as Parkinson’s disease and Alzheimer’s diseases; (v) brain tumor(s) or brain injury; (vi) unstable vital sign(s) or severe heart or renal failure; (vii) metal implants, devices, or other conditions that would forbid the application of iTBS or MRI scanning. During the study, all patients received the same form and intensity of rehabilitation treatment, including speech and language therapies, upper extremity training, gait, and mobility-related functions and activities. All patients or their legal representative(s) provided their written informed consent.

Following recruitment, each participant accepted a TMS evaluation to determine the iTBS parameters before the first session of treatment. The three-step MRI scans were conducted as follows: in the first step, the structural MRI was scanned, while in the second step, fMRI (time point 1, TP1) was scanned, and during the third step, scanning continued with fMRI (time point 2, TP2). iTBS intervention was performed between the second and third steps. No other intervention or training was provided to the participant on the same day of the above MRI scans and iTBS treatment until the study procedure was completed. The participants accepted conventional medical and rehabilitative modes of treatment during hospitalization. This study was approved by the ethics board of HuaShan hospital Fudan University (2019-336), and all procedures conform to the Declaration of Helsinki regarding human experimentation. Our trail registration number is ChiCTR-TRC-2100041936.

### 2.2. Behavioral Assessments

The Chinese version of WAB, which is a widely used clinical evaluation tool of PSA, was used to assess assessing the presence, type, and severity of aphasia for each participant. The scores on the four subscales of the Western Aphasia Battery were combined to form the aphasia quotient (AQ), which quantified the severity of each patient’s aphasia [35]. In addition, the fluency and content scores from the spontaneous speech, auditory comprehension, repetition, and naming subscales of WAB were also used to assess each participant’s language abilities.

As the motor and cognitive abilities were often impaired in the PSA patients, and importantly, because we planned to investigate how motor-area target stimulation affects the patients’ language performance, we also assessed the participants’ motor and cognitive impairments. For motor function, the Fugl–Meyer motor assessment–upper extremity (FMA-UE) was used to assess the patients’ upper limb motor function, including voluntary movement, velocity, coordination, and reflex activities. A total score was calculated combining scores of 33 items, resulting in a total maximum score of 66; higher scores indicated better motor function. For cognitive abilities, the non-language-based cognitive assessment (NLCA) was used; the scale is specifically designed and validated to assess the cognitive function of aphasic patients, which uses pictures and objects instead of language instructions and requires no speaking or writing outputs [36]. The NLCA evaluated each participants’ cognitive abilities from five domains: memory, visual–spatial abilities, attention, logical reasoning, and executive function, with a total maximum score of 80; higher scores indicate better performances. In addition, the Barthel index (BI) was used to assess each patient’s levels of activities of daily living (ADL), with a range from 0 (dependent) to 100 (independent) [36,37].

### 2.3. iTBS Procedure

**Figure 1:**
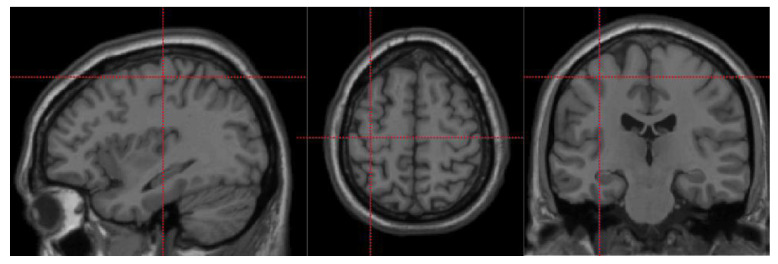
The core location of iTBS intervention (−36, −21, 58).

iTBS was delivered using a MagPro X100 magnetic stimulator (Medtronic Co, Copenhagen, Denmark) with a figure-eight coil (MC-B70). Before the first session of iTBS, the individual resting motor threshold (RMT) was examined for each subject following the procedure as follows: first, the motor evoked potential (MEP) was measured from the hemiplegia abductor pollicis brevis muscle with surface electrodes (patients with unmeasured MEP on the hemiplegic side are measured with the contralateral side) [38], and then, the “hotspot” was determined using single-pulse TMS over the primary motor area (M1) where the largest MEP was evoked; then, the single-pulse TMS was given at the “hotspot” from low intensity, and with a stepwise increasing intensity until the amplitudes of 5 out of 10 trials exceeding 50 mV; this intensity was defined as the individual RMT. The hotspot location was marked on a positioning cap for each participant, which the participant wore while receiving iTBS over the M1 area [31,39]. After determining the RMT and stimulation point for each subject, the first session of left M1 iTBS was performed with the following parameters: intensity of 80% RMT, three pulses at 50 Hz in each burst at 5 Hz, and 2 s stimulation with 8 s interval; a total of 800 pulses were delivered for one session. The precise location of the iTBS-targeted stimulation site can be found in Figure 1. The TMS machine was placed outside the MRI room, and each participant accepted consecutive MRI scanning before, immediately after the iTBS session. We focused on the immediate brain functional effects of the iTBS in this study, so only the images from the first two time points (i.e., before and immediately after the iTBS session) were included in the analyses.

### 2.4. MRI Acquisition

Patients were scanned at the Jingan Branch of Huashan Hospital, Fudan University, with a 3.0T GE MR750 scanner. The T1-weighted images were acquired using 3D FSPGR with the following parameters: matrix size = 260 × 224, FOV = 200 × 200 mm^2^, layer thickness = 1 mm, voxel size = 1 × 1 × 1 mm^3^, repetition time TR = 7800 ms, echo time TE = 5 ms, tilt angle = 12°, and number of layers = 248 layers.

The resting-state functional images were acquired in the horizontal plane by EPI echo-planar imaging TR = 2000 ms, echo time, TE = 30 ms, and tilt angle = 248 layers. Imaging sequence was acquired in the horizontal plane with the following parameters: repetition time TR = 2000 ms, echo time, TE = 30 ms, inclination = 90°, matrix size = 96 × 96, and voxel size = 3 × 3 × 3 mm^3^. The entire resting-state functional scanning procedure lasted 480 s, and a total of 240 functional images were obtained for each subject (one image every 2 s). Before scanning, each participant was instructed to keep still with eyes closed in the machine and to be relaxed with no systematic thinking.

### 2.5. Lesion Analysis

The lesions were manually segmented using ITK-SNAP tools [40] on individual structural images by inspection of the T1-weighted, T2-weighted, and FLAIR images, registered to Montreal Neurological Institution (MNI) brain using spm12 (https://www.fil.ion.ucl.ac.uk/spm/, accessed on 13 January 2021). A voxel-wise map of lesions was created by summing up all the individual lesions.

### 2.6. Preprocessing of Resting-State Functional MRI Data

The resting-state functional MR images were pre-processed following the conventional steps: (1) discarding the first 10 volumes of each image; (2) slice timing for systematic time shift; (3) head motion correction using Friston 24 parameters; (4) image reorientation for better normalization; (5) normalizing to MNI space using unified segmentation on T1-weighted images and being resliced into 3 mm × 3 mm × 3 mm resolution; (6) removing the linear signal trend; (7) regression of the nuisance variables including Friston 24 parameters, the white matter, and cerebrospinal fluid signal. Additionally, additional band-pass filtering of 0.01–0.10 Hz signal was performed for functional connectivity analyses. As spatial normalization might be influenced by the lesions, we used an enantiomorphic approach [41] to replace the lesioned brain tissue with the contralateral mirrored scans. The functional image pre-processing was all applied using the data processing assistant for resting-state fMRI, packed in the data processing and analysis for (resting-state) brain imaging (DPABI) procedures [41,42].

### 2.7. Resting-State Brain Functional MRI Data Analyses

We calculated the regional, seed-based connectivity, and graph-based measures to test the immediate functional effects of the left M1 iTBS in the aphasic brains more comprehensively. The analyses and comparisons were all restricted in a gray matter mask in accordance with the automated anatomical labeling (AAL) atlas, covering the cerebral cortex and nucleus and not including the brain stem or the cerebellum; we will refer to this mask as the “whole-brain mask” in the paragraphs below for the sake of brevity.

#### 2.7.1. Regional Functional Activity Analyses

The fractional amplitude of low-frequency fluctuation (fALFF) was calculated to measure the regional spontaneous brain activity on voxel level, which reflects the strength of spontaneous neural activities in comparison with other non-neural biological signals or artifacts [43]. The fALFF value was computed as the ratio of the summed amplitudes of signals at the low-frequency range (0.01–0.08 Hz) to the amplitudes of the entire frequency range at each voxel, resulting in a spatial map of fALFF for each subject. Then, the individual spatial fALFF maps were z-normalized in the whole-brain mask at the voxel level.

#### 2.7.2. Seed-Based Functional Connectivity Analyses

Seed-based connectivity (FC) analyses were performed to investigate the functional connectivity changes in the left M1 area, which was the site of the iTBS, with all the other voxels in the whole brain. We defined the left M1 seed as the peak activation point reported previously in a hand motion task [44] (MNI-coordinate: −36, −21, 58, Figure 1). The time courses of all voxels within a sphere of 4 mm radius around the center coordinate were averaged, and the connectivity maps were calculated by calculating the Fisher z-transformed correlation coefficient between this mean time course to the time course of each voxel in the whole-brain mask for each subject.

#### 2.7.3. Degree Centrality (DC) Analyses 

Degree centrality is a widely used graph-based nodal metric, which measures the importance of a node in a given brain network by calculating the number of other regions it connects within the network [45]. As previous studies have revealed that the functional connectivity undergoes distinguished changes within the same or between the two-brain hemisphere(s) following stroke, we calculated three voxel-wise DC metrics to measure the importance of each voxel in the resting-state brain functional network, including a whole-brain DC, DC within the same hemisphere, and DC with the contralateral hemisphere. First, the zero-lag Pearson’s linear correlation coefficients were calculated between the time courses of each pair of voxels in the whole-brain mask. Next, the individual correlation coefficients (i.e., connections) were entered into an N × N adjacency matrix, where N is the number of voxels. The voxel network matrix was thresholded by *r* > 0.25, suppressing random correlations, and was subsequently z-transformed. The three DC metrics were then calculated for each voxel by summing up all the connections it had with (i) all the other voxels in the whole brain (the whole-brain DC), (ii) all the other voxels within the same hemisphere (within-hemispheric DC), and (iii) all the voxels in the contralateral hemisphere (interhemispheric DC).

### 2.8. Statistical Analysis

Statistical analyses of fMRI data were conducted using the statistical tools of DPABI based on SPM12 and running on MATLAB. Paired *t*-tests were used to test the changes in fALFF, DC, and FC metrics between pre- and post-iTBS treatment on the voxel level. AlphaSim corrections were adopted to adjust for the multiple comparisons [20,46,47], and the probability of false-positive detection was set to *p* < 0.05.

## 3. Results

### 3.1. Demographic and Clinical Information for Stroke Patients with PSA

**Figure 2:**
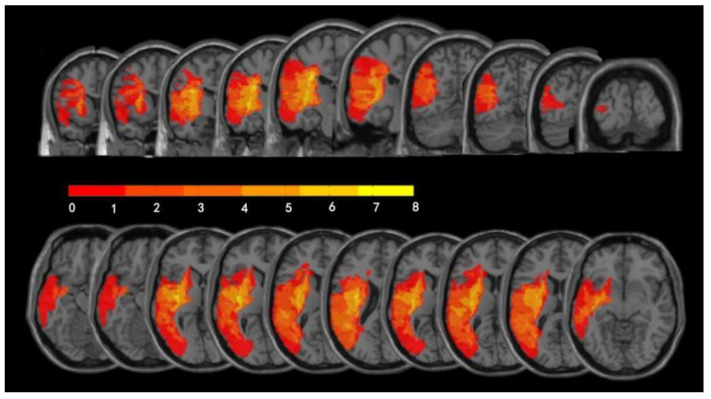
Lesion overlap map across 16 patients with PSA illustrating the distribution of lesions. The color scale in the spectrograms represents the injured brain locations’ magnitude of a frequency.

A total of 20 individuals were recruited. Four patients did not complete the MRI scanning and were thus excluded from the assessments. Eventually, 16 patients (4 females and 12 males; age (mean ± SD): 55.6 ± 11.8 years, formal education (mean ± SD): 16.0 ± 2.3 years) were included in the presented study. Table 1 shows the demographic information, behavioral assessment scores, and RMTs of the 16 patients. The individual lesion sites can be found in Table 1, and the lesion overlap map for all patients is presented in Figure 2.

### 3.2. The Immediate Effects of Left M1 iTBS on fALLF

**Figure 3:**
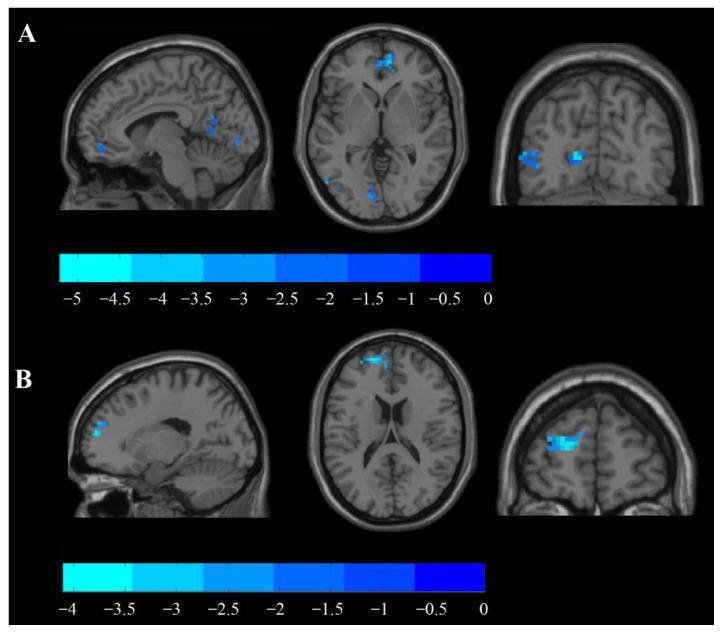
Changes in fALFF as well as FC before and after iTBS intervention: (**A**) differences in fALFF between TP1 and TP2 (paired *t*-test, *p* < 0.05, AlphaSim correction, cluster size ≥ 56 voxels); (**B**) differences in FC with left precentral gyrus (−36, 57,18) between TP1 and TP2 (paired *t*-test, *p* < 0.05, AlphaSim correction, cluster size ≥ 129 voxels). The blue concentration represents the degree of decreased regional fALFF or FC values. AAL: automated anatomical labeling atlas, BA: Brodmann area, R: right, L: left.

After the left M1 area iTBS was performed, two clusters exhibited decreased fALLF values, as identified by the paired sample *t*-test in the brain regions including the right paracingulate gyrus (BA10) and the left intracalcarine cortex (BA17), respectively (*p* < 0.05, with AlphaSim correction; Table 2, Figure 3A).

### 3.3. The Immediate Effects of Left M1 iTBS on Its Functional Connectivity

Immediately following the left M1 iTBS, the functional connectivity between the hand area of the left M1 and the left precentral gyrus, as well as the left frontal pole, was found to be significantly decreased, as tested by paired two-sample *t*-test (*p* < 0.05, with AlphaSim correction; Table 3, Figure 3B).

### 3.4. The Immediate Effects of Left M1 iTBS on DC

**Figure 4:**
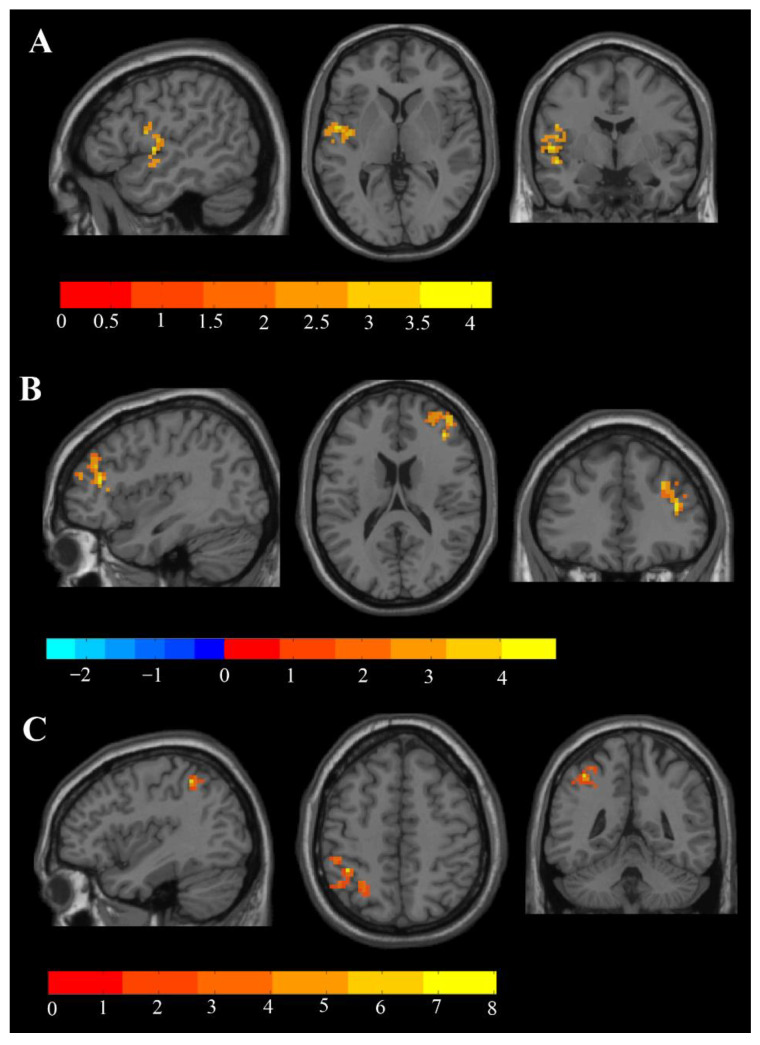
Changes in DC before and after iTBS intervention: (**A**) differences in interhemispheric DC between TP1 and TP2 (paired *t*-test, *p* < 0.05, AlphaSim correction, cluster size ≥ 169 voxels); (**B**,**C**) differences in DC of whole grey matter between TP1 and TP2 (paired *t*-test, *p* < 0.05, AlphaSim correction, cluster size ≥ 142 voxels). The blue areas represent the regions that have decreased DC values, while the yellow areas represent the regions that have increased DC values.

The three DC metrics showed different changes after the left M1 area iTBS in our PSA patients. For the whole-brain DC, one cluster in the right middle frontal gyrus and one cluster in the left middle frontal gyrus showed significant elevation from pre- to post-intervention (*p* < 0.05, AlphaSim corrected). Interhemispheric DC values were also revealed to be increased in the left central opercular cortex (*p* < 0.05, with AlphaSim correction), while no significant change was found for within-hemispheric DC (Table 4, Figure 4).

## 4. Discussion

To the best of our knowledge, iTBS can modulate cortical excitability and may serve as a potential tool for neuroplasticity of impaired motor, language, and cognition brain areas [48,49,50,51]. This study focused on immediate effects and changes of brain activity in patients with PSA, modulated by iTBS. After a combination of iTBS and fMRI, our results reveal that iTBS acting on the M1 area of the affected hemisphere can cause altered cortical excitability and functional connectivity of different regions, which may further lead to orientative neural plasticity in language- and cognition-related functional areas of the brain.

iTBS can increase ipsilesional cortical excitability and has been increasingly used in patients with stroke. Combining fALFF, FC, and DC of rs-fMRI datasets before and after a brain stimulation protocol allowed us to map the changes throughout the whole brain induced by iTBS, instead of only looking at the single change of excitability. We found that after 200 s iTBS intervention, fALFF, DC, and FC all changed to different degrees, which is an indication of the immediate effects of iTBS and is promising for neural plasticity after PSA. Such changes that occur in the neural network of the patient’s brain for a long time will produce qualitative changes and cause behavioral progress. Regarding fALFF, we found decreased fALFF values in two clusters in the right frontal and left parietal lobes (Figure 3). The fALFF value stands for spontaneous neuronal activity in brain regions by directly observing the magnitude of baseline changes in the blood oxygenation level-dependent (BOLD) signal of functional brain activity [52]. After the iTBS intervention, the fALFF value in the right frontal part was reduced, suggesting that iTBS attenuated neuronal activity in the contralateral brain. According to the interhemispheric inhibition theory [53,54], the affected hemisphere is inhibited for a certain period after brain injury, while the healthy hemisphere is excited, a condition that is not conducive to functional recovery after stroke. iTBS acting on the left impaired M1 area can inhibit brain activity in the right medial frontal lobe, and some studies [55,56] have shown that right frontal lobe excitability is closely related to the recovery of language status after stroke. For example, A TMS study indicated that non-fluent aphasia after stroke is due to an imbalance in the functional network of the language brain in the bilateral hemispheres [57,58]. Further, 1 Hz of rTMS acting on the right Broca suppressed the excitatory state of this region and promoted language function recovery, especially naming and spontaneous speech ability in patients [59]. The altered right frontal lobe is close to the mirror area of the dominant hemisphere language area (Broca); therefore, this change might promote the recovery of language function and cognitive function in patients with PSA. It was confirmed that the fALFF value was positively correlated with cognitive evaluation scores [60], while the parietal fALFF value in the ipsilateral hemisphere decreased, suggesting that the immediate effect of iTBS might not improve the excitability of brain neurons in cognition-related parietal regions.

DC refers to the number of connections between a voxel and other voxels in the whole brain, and the centrality of a voxel in the whole brain is evaluated by the change in the number of connections. DC value can make full use of the whole brain signal and can avoid subjective selection of seed points, which is a more reliable fMRI analysis method. To find the immediate effect of iTBS in the whole brain and hemispheres accurately, we observed from three perspectives: whole brain, interhemispheric, and within hemispheric. In whole-brain gray matter analysis, the DC value in the right cortical area BA45 and the left BA40 area significantly increased after iTBS (Figure 4). Rao et al. [61] confirmed that after 7 days of rTMS in the affected Broca area rTMS intervention, the patients’ DC values in both Broca areas increased significantly and gradually approached the normal level compared with the preintervention. Meanwhile, the patients’ spontaneous speech and auditory comprehension functions were significantly improved compared with baseline. Our results indicated that iTBS, as a special mode of TMS, may have similar effects. In addition, consistent with our initial hypothesis, stimulation of the affected M1 area achieved such effects, which manifests that there may be some functional connection between the M1 area and the contralateral Broca’s area. This phenomenon has been found in some studies [62]. Similarly, the role of the left BA40 area as part of Wernicke’s area, which is traditionally considered to be the comprehension of language, is an innovative finding of our study. From the within-hemispheric perspective, we failed to observe changes in DC value in some brain regions. However, from the interhemispheric point of view, the significant growth of DC was found in the left BA48 area, which is similar to the results observed from whole-brain gray matter. The seed-to-seed FC intensity between the stimulation target and left BA10 was reduced (Figure 3). It is believed that the BA10 area has a close relationship with cognitive function [62,63,64], which may suggest that we should focus on cognitive functional changes after iTBS-stimulated area M1. Furthermore, the study by Hara et al. [26] found that the lateralization index of BA10 was closely related to the outcome of speech dysfunction treatment after speech training and TMS intervention.

There are some limitations to our study. Firstly, our study was designed as a single-arm study and without a proper control group; thus, results can only be compared between preintervention and postintervention within a group, which made it difficult to diminish the effects caused by confounding factors beyond the intervention of concern and may have caused uncritical results. Second, due to certain technical limitations, we did not perform further analysis of brain network connectivity, which will be completed in our next study. Third, the study adopted a traditional localization paradigm for the localization of the M1 region and did not use a precision navigation localization method. However, we performed an accurate individualized localization cap for each patient with a homogeneous localization pattern. We selected the MEP maximum amplitude location and obtained the same location after positioning by two specialized physicians before using it as the final stimulation site. In a future study, we will apply TMS navigation technology to further improve the accuracy of this study.

## 5. Conclusions

To the best of our knowledge, few studies have characterized the brain regional dynamics induced by iTBS manipulation on the left M1 area in patients with PSA. The fALFF and DC measurements consistently demonstrated a significant immediate iTBS effect around language- and cognition-related regions, while FC showed iTBS effect was prominent between the affected M1 and frontal pole region. From this perspective, iTBS on the left M1 region may be a promising rehabilitation tool to enhance language and cognition rehabilitation for patients with PSA. In the future, more clinical randomized controlled trials of iTBS are needed to verify the long-term therapeutic effect and to evaluate the progress of clinical behavior and the brain mechanism.

## Figures and Tables

**Table 1:** Demographic and clinical information of the participants.

Demographic Information							Functional Assessment					
Patient Code	Age (Years)	Sex	Education (Years)	TSI (Months)	Type of Stroke	Lesion Location in LH	Handedness	WAB-AQ	FMA-UE	BI	NLCA	RMT
P1	70	F	12	3	Ischemic	IFG, STG, BG	R	3	25	50	5	60
P2	40	M	16	2	Ischemic	IFG, PreCG	R	90.2	22	90	75	46
P3	58	F	12	2	Ischemic	IFG, BG	R	60	45	75	65	52
P4	68	M	12	8	Ischemic	BG, MTG, AG	R	61.3	37	35	61	43
P5	65	M	12	7	Ischemic	IFG, BG	R	62.4	9	40	45	40
P6	41	F	16	2	hemorrhage	AG, BG	R	89	25	90	75	34
P7	58	M	9	7	Ischemic	AG, PreCG	R	95.8	21	60	76	48
P8	69	M	12	6	Ischemic	IFG, BG	L	76.1	34	35	57	43
P9	43	M	15	8	Ischemic	IFG, PreCG	R	95	27	50	79	50
P10	60	M	9	9	Ischemic	STG, BG, IFG	R	7	7	10	15	60
P11	68	M	12	6	Ischemic	IFG, PreCG	R	85.6	24	60	70	34
P12	42	F	15	4	hemorrhage	STG, BG	L	18.1	24	55	53	76
P13	62	M	9	5	Ischemic	AG, BG	R	71.2	50	90	73	41
P14	41	M	12	3	hemorrhage	IFG, BG	R	68.5	40	60	65	46
P15	62	M	12	5	hemorrhage	STG, BG	R	45	33	50	60	65
P16	42	M	15	5	Ischemic	IFG, BG	R	69	37	65	72	60

Abbreviation: TSI, time since injury; WAB-AQ, Western Aphasia Battery aphasia quotient; FMA-UE, Fugl–Meyer assessment of upper extremity; BI, barthel index; NLCA, non-language-based cognitive assessment; RMT, resting motor threshold; LH, left hemisphere; IFG, inferior frontal gyrus; BG, basal ganglia; preCG, pre-central gyrus; MTG, middle temporal gyrus; AG, angular gyrus; STG, superior temporal gyrus.

**Table 2:** Significant differences in regional fALFF between TP1 and TP2.

Brain Region (AAL)	Brain Region (BA)	Cluster Size (Voxels)	Peak MNI Coordinates (mm)			T Value
Paracingulate gyrus (R)	BA10_R	56	9	51	0	−5.32
Intracalcarine cortex (L)	BA17_L	81	−12	−72	9	−4.65

**Table 3:** Significant differences in the ROI-to-ROI functional connectivity between TP1 and TP2.

Seed Based Functional Connectivity	BA	Cluster SIZE (Voxels)	Peak MNI Coordinates (mm)			T Value
Frontal pole (L)	BA10_L	129	−18	57	18	−4.11

**Table 4:** Significant differences in regional DC between TP1 and TP2.

Brain Region	BA	Cluster Size (Voxels)	Peak MNI Coordinates (mm)			T Value
Whole grey matter						
Frontal pole (R)	BA45_R	142	39	36	18	4.83
Superior parietal lobule (L)	BA40_L	158	−39	−45	51	8.07
Interhemisphere						
Central opercular cortex (L)	BA48_L	169	−51	−3	3	4.19

## Data Availability

Data are available on reasonable request.
